# Determination of Buckling Behavior of Web-Stiffened Cold-Formed Steel Built-Up Column under Axial Compression

**DOI:** 10.3390/ma15092968

**Published:** 2022-04-19

**Authors:** Muthuraman Mohan, Anuradha Ramachandran, Mugahed Amran, Aleksey Borovkov

**Affiliations:** 1 Department of Civil Engineering, University VOC College of Engineering, Thoothukudi 628008, India; 2 Department of Civil Engineering, SNS College of Technology, Coimbatore 641035, India; anuradhastalin@gmail.com; 3 Department of Civil Engineering, College of Engineering, Prince Sattam Bin Abdulaziz University, Alkharj 16273, Saudi Arabia; m.amran@psau.edu.sa; 4 Department of Civil Engineering, Faculty of Engineering and IT, Amran University, Amran 9677, Yemen; 5 Institute for Advanced Manufacturing Technologies, Peter the Great St. Petersburg Polytechnic University, 195251 St. Petersburg, Russia; borovkov@compmechlab.com

**Keywords:** stiffened section lipped built-up columns, back-to-back battened section, DSM, FEM

## Abstract

The practice of utilizing cold-drawn steel for structural and non-structural elements has expanded nowadays due to it being lighter in weight, economic section, desirable in fabrication, and its preferred post-buckling behavior over hot rolled sections. The cold-drawn steel section back to the back-lipped channel section has a wide application as a structural member. The fasteners are provided at regular intervals for the long-span structure to prevent individual failures. This study is concerned with the inadequacy of research addressing the behavior of built-up columns. The relevant built-up column section is chosen based on the AISI-S100:2007 specification. Thirty-six specimens were designed and tested by varying web, flange, lip dimensions, spacing between the chords, and battened width experimentally subjected to an axial compression. Comparing 36 experimentally buckled specimens with the model generated by Finite Element Method accompanied with ASI-recommended two direct strength methods (DSMs). The DSM comprises the step-by-step procedure incorporated with the elastic, critical, and global distortional interaction. Based on the performed reliability analysis, such as the experimental, analytical, and theoretical studies, the failure load, buckling mode, the economic section, and design rules were proposed. Four suitable sections were selected from the proposal, and the validation study was carried out. From the validation study, experimental values were found to be 1.072 times the FEM values, and DSM values were found to be 0.97 times the FEM values. Based on the significant findings of this study, the proposed design recommendation and the corrected value for DSM are suitable for designing back-to-back stiffened columns.

## 1. Introduction

In modern construction, the usage of cold-formed steel sections is needed. The cold-formed steel structural members such as beams, columns, and joists are widely used. The main advantage of the cold-formed steel (CFS) is the post-buckling behavior where the hot rolled steel section fails early during axial loading in the single section member. Hence, the area is modified by placing two-lipped chords at a certain distance. Those two chords are connected back-to-back by batten with bolted connection to perform like a single member during loading. The failure mode of the column was influenced predominantly by the local buckling mode. However, there are some limitations in predicting the column’s buckling mode by varying the specifications such as the size of the battened plate, dimensions of the section, and the slenderness ratio during the experimental investigation. There is a need for conducting a parametrical study. So that area chosen for our research was selected from the codal specification in AISI-S100:2007 [1]. The desired section was tested and validated with Finite Element Analysis (FEA) and Direct Strength Methods (DSMs).

Salem et al. [2] investigated I-slender sections with different members by varying slenderness ratios and spacing between the battens. Nonlinear analyses using FEM Software and the strength equation proposed. Kalochairetis and Gante [3] conducted a numerical and analytical investigation for the laced built-up column to determine the collapse load and predicted that the member’s capacity decreases by 50% due to imperfections. Based on works reported by Kandasamy and Jayagopa [4], CFS-lipped channel beams subjected to a restrained condition whose lip thickness is about 1.5–2mm will be considered the optimum size for the flexural-torsional buckling. Rondal and Niazi [5] proposed a new design rule by conducting a parametric study on C-type stitches and batten elements as connecting members for channel profiles. By varying the slenderness ratio in the hot rolled steel compression members [6], the experimental and analytical investigation validated American Institute of Steel Construction specification for designing the built-up member. Roy et al.’s [7,8,9] work on back-to-back battened built-up sections showed that fasteners between two built-up areas and the region’s thickness influence the load-carrying capable built-up units. Roy et al. [9] revealed the specimen chosen for study based on AS/NZS 4600 [10] and AISI-S100 [1]. Sani et al. [11] worked on C sections with v notches. The most extensive V notch depth has a greater load-carrying capacity than the regular c section, and the experimental results were agreeable with the Eurocode 3—Part 1 (EC 3-Part 1) specifications [12]. Anbarasuet al. [13] ensured the necessity for choosing bolted connections and ties along the lateral direction in suitable spacing. The fastener influenced a CFS section column for the structural members, such as a beam and column joint. Because the specimen is of pined ends will provide additional tensile strength to the member. The proposed design rule ensures the combined behavior of the assembly. By carrying over an experimental investigation on battened four equal slender angle columns and found that the member’s slenderness ratio influenced the column’s failure mode [14,15]. The direct strength methods approach has been made for Z sections connected side-to-side by Georgieva et al. [16,17]. It is shown that the Z section will provide post-buckling strength during distortional and flexural buckling. He recommended the appropriate design for spaced built-up columns subjected to axial loading based on EC 3-Part 1 [12] and AISI recommendations [1].

Anbarasu et al. [18] performed a parametric study on stiffened, lipped, back-to-back connected Built-up sections using FEM and DSM analysis and found the conservative DSM method for predicting the failure load. Muthuraman et al. [19] had validated Ting and Lau’s experimental work [20] and performed a parametric study by varying the slenderness ratio, batten width, and spacing between the chords. Based on the research, the slenderness ratio was up to 60, the DSM approach was conservative, and the section was unstable above the slenderness ratio of 60. Schafer and Peköz [21] justify that incorporating residual stress and geometric imperfection will influence the load-carrying capacity on finite element modeling (FEM) analysis.

Kankanamge and Mahendran [22] investigated a CFS beam subjected to uniform bending and found lateral-torsional buckling. The numerical investigation predicted new design rules for lateral-torsional buckling of CFS lipped channel beams design using EC 3-Part 1 [12]. Hajirasouliha et al. [23] proposed the optimized technique for designing lipped channel sections and mentioned the improvement in lateral strength of the laterally braced and unbraced column by 75% subjected to axial loading considered during the design in this study. Dinis and Camotim [24] investigated column structural behavior against local Distortional buckling of the hat, zed, and rack-shaped sections and validated the novel DSM method design approach. The work describes the local-distortional interaction that influences the failure mode in load-carrying capacity. The suitable selection of the lipped channel section was based on the recommendation of Hajirasouliha, and Becque [25] worked on the CFS lipped channel section and studied the interaction of local and overall flexural buckling mode. For the optimized lipped channel section, their ultimate load-carrying capacity is 19% more than the commercially available section and was agreeable with the EC 3-Part 1 [12]. Aswathy and Kumar’s [26] study on stiffened and unstiffened lipped channel sections shows the decrease in stiffness or depth of the lip will increase the chance for distortional buckling. The study gave clear information about the distortional buckling in the stiffened lipped channel section during axial loading and predicted that the limiting case for distortional buckling is a partially stiffened element.

The work progresses by performing the validation study similar to the observation reported by Kumar and Kalyanaraman [27]. That work shows the strength of CFS lipped channel compression members reducing its power by the member interaction between the buckling modes during axial loading. This study demands the need for the DSM approach for individual buckling modes to predict the accurate load and buckling interaction. In this study, short columns are selected for experimental study rather than slender columns based on Numerical investigation performed by Dar et al. [28]. The work proves that the ultimate capacity of the column goes on reduced by an increase in toe–toe spacing and slenderness ratio, which even affect the behavior of battens in the built-up column during loading. Dar et al.’s [29] and Vijayanand and Anbarasu’s [30] validation studies predicted that North American Specification and Euro standards are found to be unconservative by 15 to 30 %; the slenderness ratio needs to be restricted by 75 to avoid lateral drifting and large lateral displacements. The reason for choosing the open section rather than the closed section is based on the main findings of the research reported by Kherbouche and Megnounif [29]. The nonlinear finite element analysis is performed on both open and closed channels connected by battened plates. The study showed that the stability of the column is influenced by spacing between the channels ‘web to length ratio. The open sections are found to be conservative, and failure is influenced by local buckling. Moreover, the closed sections failed by global buckling, and the results are found to be un-conservative. The limitation of the spacing between the chords is chosen based on the recommendation of Vijayanand and Anbarasu’s [31] validation study stated that the increase in spacing between the chords and varying slenderness ratio from 20 to 120 would influence column strength and found the conservative DSM method predicted the column strength. Anbarasu’s [32] work on four lipped channel sections shows that a column with a lower chord slenderness ratio significantly affects the axial load compression. In that work, the predicted FEA models were agreeable with the experimental test results.

Zhang and Young [33] conducted a CFS column investigation. It is revealed that stiffener facing inwards has more buckling strength against axial loading and defines the need for conducting regression line analysis to predict the relationship between the failure load of the FEA and DSM approach for the validation study. The modified value of the failure load using DSM has been adopted and applied for numerical investigation from the validation study. The concept of the corrected value of failure load is adopted from Gunalan and Mahendran [33] and preceded. The reason behind choosing the pinned end connection is based on the recommendation of Martins et al. [34] regarding the investigation subjection to flexural-torsional buckling. The work shows good performance of pinned end condition under warping, and NAS prediction for failure load under DSM is found to be closely agreeable by modifying the equation using the reduction factor. The pre-analysis using Generalized Beam Theory at the University of Lisbon (GBTUL) was referred to by Martins et al. [35], and Cava et al. [36] reported pre-analysis works on cold-formed steel columns with a lipped channel to find out the local–distortional interaction effects on its design and behavior. The study reveals the web triggered the L-D interaction. The failure load availed from L-D interaction was quite agreeable with DSM results and the need for carrying over-generalized beam theory for post-buckling analysis. Manikandan and Arun [37] demand the provision of intermediate stiffeners, and the ratio of center to center of the spacer plate to the length of the column will influence the torsional rigidity for the partially closed sections. The values of DSM were found to be conservative. Ref. [38] detailed learning reveals the need for conducting regression line analysis for the CFS section subjected to axial loads. From [39,40] performed work outcomes, the numerical investigation and strengthening techniques are adopted for effective section design Roy, K. et al. [41] suggestions are incorporated into the FEA analysis and modeling. Liang, H. et al. [42] is a review on CFS members, which ensures better structural and thermal performance for the chosen section.

Anbarasu et al. [18] and Muthuraman et al. [19] work on the unstiffened built-up section by parametric validation and numerical analysis. This work advances by converting the unstiffened section in Muthuraman and Anuradha [19] to a stiffened section by providing intermediated stiffener in both sections. Based on the limitation of AISI-S100 [1], six different cross-sections are experimentally and analytically tested in this study. In a repetition pattern by varying the slenderness ratio and batten width, 36 specimens were analyzed. A parametric study was carried out from the recommendation proposed by the validation work. The parametric study comprises about two different sections. By varying its battened width and slenderness ratio, a total of 40 specimens were analyzed using FEM and compared with DSM results. Based on the outcomes, a suitable design method is said to be proposed to predict the failure load for the CFS web-stiffened column using the DSM.

## 2. Materials and Methods

### 2.1. Selection of Specimen

Based on the AISI-S100 [1] recommendation in Table 1, six models on three times repeatability patterns, for a total of 36 web-stiffened lipped channel sections, were tested by varying geometrical specifications, as shown in Table 2. The channels are connected by battened plates using self-drilling screws as per AISI-S100 [1] guidelines. The span, web, flange, and lip thickness dimensions varied and underwent local bucking, distortion bucking, and overall bucking during axial loading. The selected specimen was analyzed based on the GBTUL code provisions [37]. Based on previous work reported by Muthuraman et al. [19], the validation study on unstiffened built-up sections, the selected section is modified to a stiffened built-up section by varying the web, flange, and geometrical specification. Finally, experimental, analytical, and theoretical work is performed.

### 2.2. Properties of the Specimen

The selected specimen is a web-stiffened lipped channel section with a thickness of around 2 mm, whose percentile value of elongation, failure stress, and maximum yield stress shown in Table 2 agree with the ASTM C 370 [38] standard specification. Since the cold-formed steel members will tend to yield by that failure load can be found, and the failure stress of the section can be found by the offset method.

**Figure 1:**
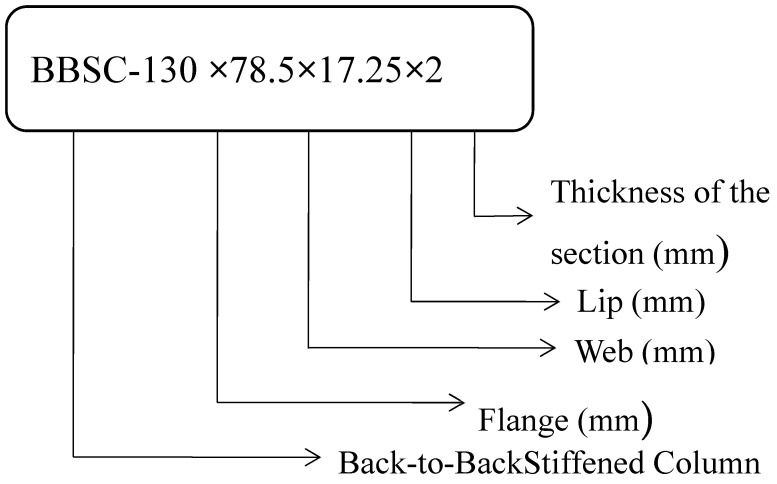
Description of the specimen.

**Figure 2:**
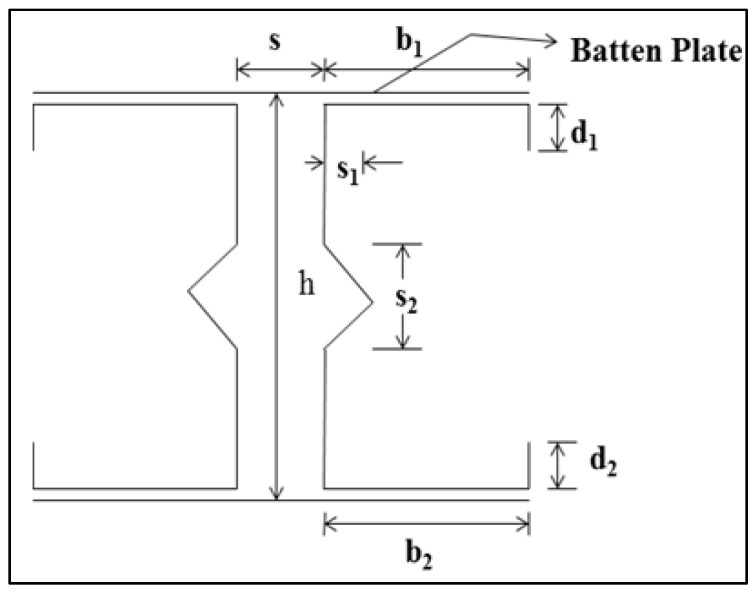
Specimen details.

Based on [1] the limitation, the specimen studied satisfies the specification for tensile stress and failure stress, which vary from 289 to 581 N/mm^2^ and 72 to 482 N/mm^2^, respectively. Similarly, the proportion of tensile strength to the yield strength ranges from 12 to 27. The detailed geometrical specifications and properties, such as failure stress, maximum stress, percentage of elongation, and modulus of elasticity are stated in Table 2. The specification for the individual section is mentioned in Table 3. The labelling is done as shown in Figure 1 and Figure 2.

### 2.3. Validation of the Selected Specimen with GBTUL

Based on the GBTUL codal specification, the single unstiffened section is selected, and Anbarasu and Murugapandian [43] performed validation work as a reference to the material properties. From the analysis of the section, Figure 1 shows the change in critical buckling for the column BBSC-20-105-2–1. Usually, the column fails by three variants of failure mode depending upon the span. For a length L < 600 mm, the column will exhibit local buckling, the distortional buckling occurs where the length L lies in between 600 < L <1960 mm, and the flexural buckling occurs when L < 1960 mm. It shows the column’s buckling behavior (local, global, and flexural) for their corresponding length.

**Figure 3:**
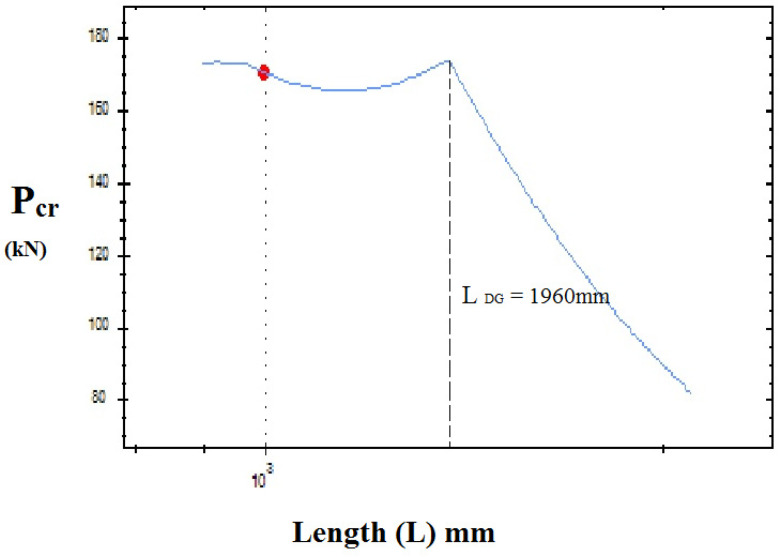
Buckling curve for the critical load P_cr_ versus span of the column.

**Figure 4:**
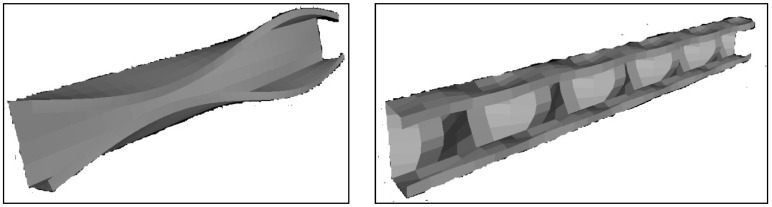
Local buckling mode and global buckling mode.

The buckling curve diagram indicates that for the 1960 mm accord and with D-G critical loads of *P_crd_* = 170 kN with *P_cre_* = 178 kN for the chosen section, the critical load analysis shows that the failure mode changes from distortion buckling way to global buckling for 1960 mm, as shown in Figure 3. The D-G interaction influenced the post-buckling behavior of the column, as shown in Figure 4. Hence, for the experimental work, the single chord section is placed back-to-back and connected with a batten to improve the post-buckling behavior under loading. To calculate the effective length, the actual distance summed off with both pinned ends (i.e.) 1960 + 37 mm for a non-loading part at the bottom end + 25 mm loading part at the top end equals an effective span of 2022 mm. Specifications in Table 3 (1,2,3) indicate the repetition of the specimen to obtain accurate results.

### 2.4. Testing of Specimen

**Figure 5:**
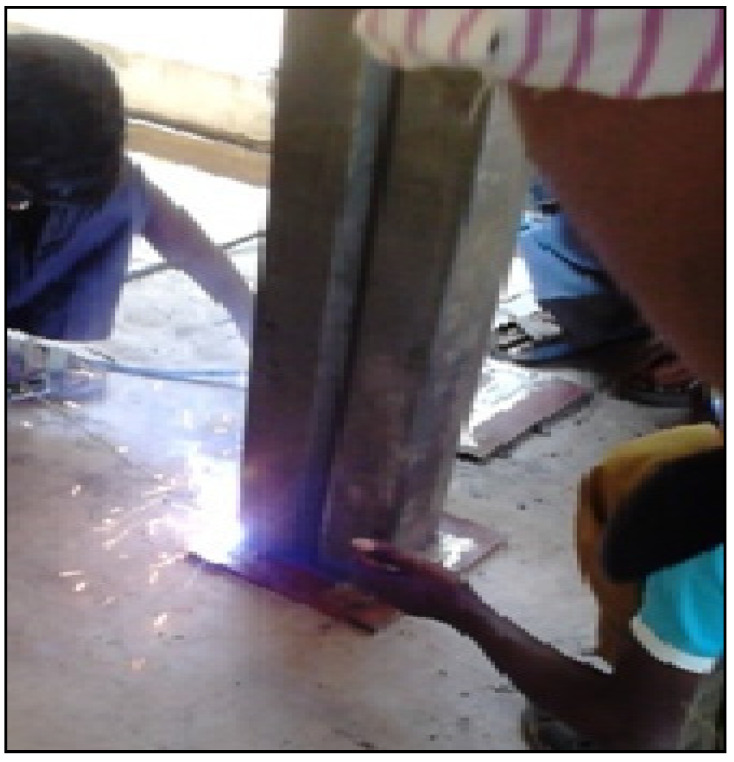
Specimen fabrication.

The sheets used for testing are of 2 mm thickness cold rolled sheets made up of pressed brake form as per AISI-S100 [1] specification. The sheet profiles of horned edges whose edge radius is negotiable. The corners of both sides connected by the plates are made of carbon of 10 mm, as shown in Figure 5. To provide uniform load transmission during axial loading and avoid confined warping at the ends; for achieving the pinned end condition, round-shaped 60 mm balls between the endplates, along the top and bottom of the flat plate. The experimental setup is chosen from Anbarasu and Murugapandian’s [39] investigation of cold-formed steel specimens.

**Figure 6:**
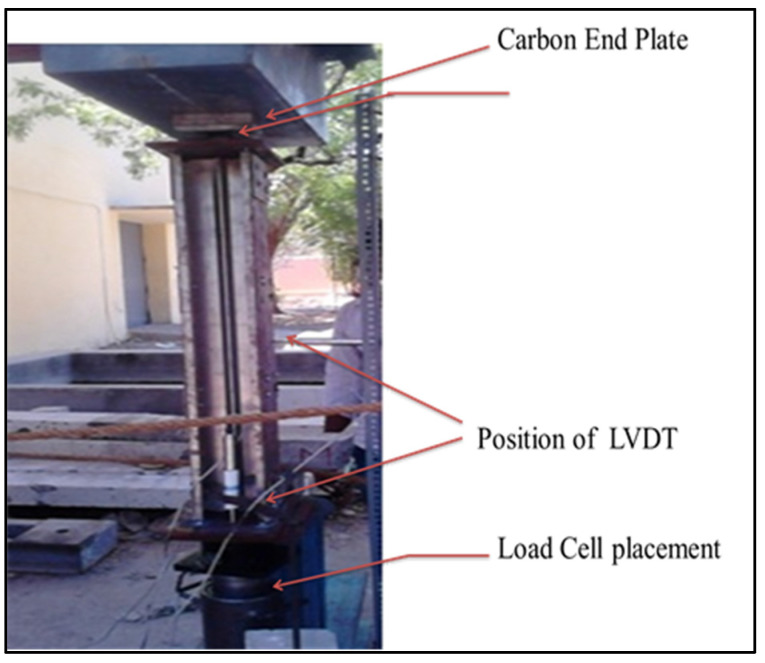
Experimental set up.

Before loading, the LVDT is fixed in a specific place to determine the deflection at all three axes. It includes placing one at half the span of the column, the second one at the middle of the web, and the third one at the center of the flange. The applied loads are captured by the transducer at suitable intervals with the help of a data logger, as shown in Figure 6.

**Figure 7:**
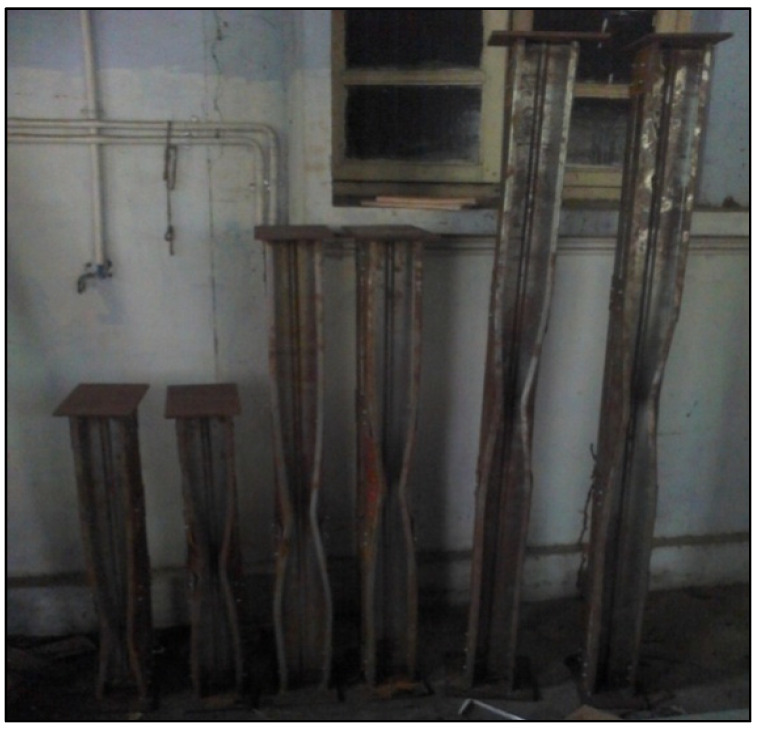
Some of the buckled specimens after load subjection.

The loading has been started incrementally by initially applying 2–5 kN as pre-load to ensure the specimen end is well connected with the endplates. The gradual loading is conducted by employing jack works hydraulically until the column achieves the failure. The maximum loads and their failure modes obtained from the test are shown in Table 4. All the specimens are found to fail from the trial by the combination of local and distortional buckling (local and flexural buckling), as shown in Table 4. The buckled models after loading are shown in Figure 7.

### 2.5. DSM (Direct Strength Method) Approach

The AISI-S100 [1] specification provides an effective method for finding the maximum load-carrying capacity of the stiffened cold-formed steel column subjected to local distortion overall buckling. Changing the slenderness ratio and providing the fasteners at regular intervals are mentioned in AISI-S100 [1] Recommendation D 1.2 described in Equations (1)–(7).

(1) $${\left(\frac{\mathrm{KL}}{r}\right)}_{m}=\sqrt{{\left(\frac{\mathrm{KL}}{r}\right)}_{O}+{\left(\frac{a}{r_{i}}\right)}^{2}}$$

where (KL/r)_O_—total slenderness ratio of the specimen; K—effective length of the member;

L—unbraced member length;

a—spacing between intermediate fastener or spot weld;

r_i_—minimum radius of gyration of the full unreduced cross-sectional area of an individual shape in the built-up member.

For the DSM design approach for calculating the axial load-carrying capacity of the member, the minimum value of the nominal member capacity, such as local buckling (*P_nl_*), distortional buckling (*P_nd_*), and flexural torsional or torsional buckling (*P_ne_*).

*P_n_*—Minimum (*P_nl_*, *P_nd_*, *P_ne_*).

The nominal axial resistance *P_ne_* for the flexural and torsional buckling is calculated as shown below:

(2) $$P_{ne}={\left(0.658\right)}^{\lambda c^{2}})P_{y}\;\mathrm{for}\;\lambda_{c}\;\leq \;1.5\;$$

where

(3) $$\lambda_{c}=\sqrt{P_{y}/P_{cre}}\;\mathrm{and}\;P_{y}=a\;f_{y}P_{y}$$

(4) $$P_{ne}=\left(\frac{0.877}{{\lambda^{2}}_{c}}\right)P_{y}\;\mathrm{for}\;\lambda_{c}\;\leq \;1.5\;$$

*f_y_*—stress due to local buckling; *P_cre_* represents the minimum critical elastic buckling load in flexural and torsional buckling;

a—Total cross-sectional area;

*P_y_*—squash load.

For the local buckling, the nominal axial resistance *P_nl_* can be find out by the following equation.

(5) $$P_{nl}=P_{ne}\;\mathrm{for}\;\lambda_{l}\;\leq \;0.776,$$

(6) $$P_{nl}=\left[1-0.15{\left(\frac{P_{crl}}{P_{ne}}\right)}^{0.4}\right]{\left(\frac{P_{crl}}{P_{ne}}\right)}^{0.4}P_{ne}\;\lambda_{l}=\sqrt{\frac{P_{ne}}{P_{crl}}}\;$$

where:

*P_ne_*—obtained from Equations (3) and (4);

*P_crl_*—critical elastic column buckling load.

For calculating the nominal axial resistance *P_nd_* under distortion, buckling can be calculated as follows:

(7) $$P_{nd}=\left\{\begin{array}{l} P_{y}\\ \left[1-0.25{\left(\frac{P_{crd}}{P_{y}}\right)}^{0.6}\right]{\left(\frac{P_{crd}}{P_{y}}\right)}^{0.6}P_{y} \end{array}\right\}\;\lambda_{d}=\sqrt{\frac{P_{y}}{P_{crd}}}$$

where:

*P_crd_*—Critical Elastic buckling load of the column under distortion mode;

*P_crd_*—*Af_od_*, *f_od_* represents stress under elastic buckling of distortional mode.

From the above equations, the desired failure loads under local distortion and flexural buckling have been found, and taking the minimum of them as the failure load under DSM (P_DSM_) will be considered for the validation study.

### 2.6. Modeling of Specimen Using FEM

#### 2.6.1. Finite Element Modeling

The finite element method is carried out for all the specimens to perform a numerical investigation of the experimentally tested model. The elastic and nonelastic mode behavior study deal with the analysis performed in ABAQUS [44]. The modeling was carried out by generating a stiffened back-to-back section. The properties of the FEM generated model are chosen from Table 2 of the properties of the experimental model for validating the experimental results with FEM results. As Schafer (23) stated, the load-carrying capacity of the area was influenced by residual stresses while analyzing a channel section. After carrying over the linear analysis, nonlinear analysis was only performed after feeding the imperfection details and residual stresses. Finally, the plotted load-carrying capacity versus the shortening graph gives the maximum load-carrying ability of the section for parametric validation. 

#### 2.6.2. Incorporation of Material Parameter

As reported by Anbarasu et al. [18], the approach for the model in the material comprises choosing the type of the element and size of the mesh. The ABAQUS model classified several nodes as S4R5 thin segments with six degrees of freedom for an individual node for the convergence studies. Initially, for the selected specimen, the length to the breadth ratio is said to be chosen as one for obtaining accurate outcomes and reducing the analysis duration. The minimum area of the mesh should be 100 mm^2^ (10 mm × 10 mm) for the practice. The analysis carried a nonlinear analysis on Stiffened sections with lip edges. The global imperfection quantity is identified from one of the thousandth spans from the middle portion of the column. Both local and global imperfections are (0.006 W × T) and (1.0 t), respectively. Therefore, the first minimum Eigen value obtained from the analysis is taken as the overall failure mode for the flexible approach. The determined value is said to be superposed to find out the inelastic performance of the section.

#### 2.6.3. Selecting the End Condition and Loading

**Figure 8:**
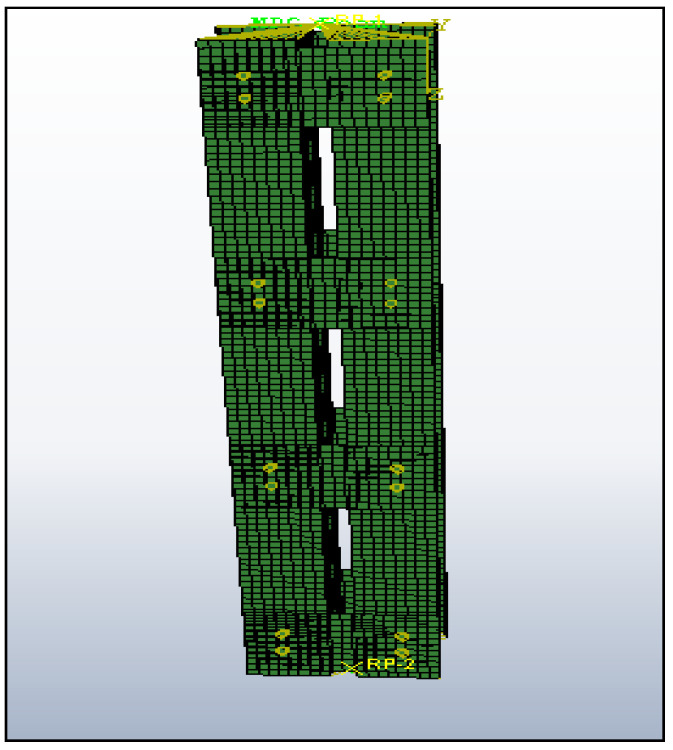
Individual nodes connected with MPC constraints.

**Figure 9:**
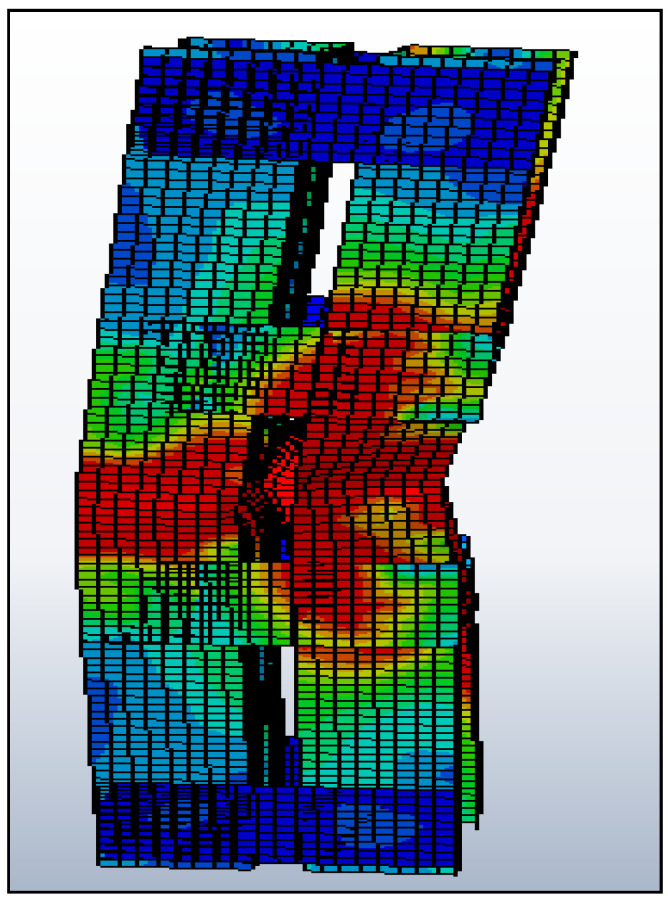
Buckled column under nonlinear analysis.

The specimen loading load ends are restricted against translation and rotation along the *x*, *y* and *z* axes, respectively. As shown in Figure 8, all nodes need to connect with an individual tie called MPC (multi-point constraint). Because the edges have to behave as a single one during loading, the generated MPC point must be at the center of gravity point of the geometrical section. As mentioned in Table 2, the specifications (including linearity of the specimen and geometric of the unit) and end conditions are similar to the experimental setup and other attributes that proved Roy et al.’s [7] claim that the in-built stress has less impact to be omitted. The applied load must pass through the C.G. points of the specimen. The loading pattern must be in the form incremental manner using the RIKS method, and the buckling of the column was discovered, as shown in Figure 9.

## 3. Results and Discussion

### 3.1. Validation of Experimentally Tested Specimen with FEM and DSM

The built-up section subjected to axial loading undergoes lateral deflection and buckles based on the load incrementation. Based on the cross-section, slenderness ratio, and material properties, load-bearing behavior varies along with failure modes, as discussed in detail.

**Figure 10:**
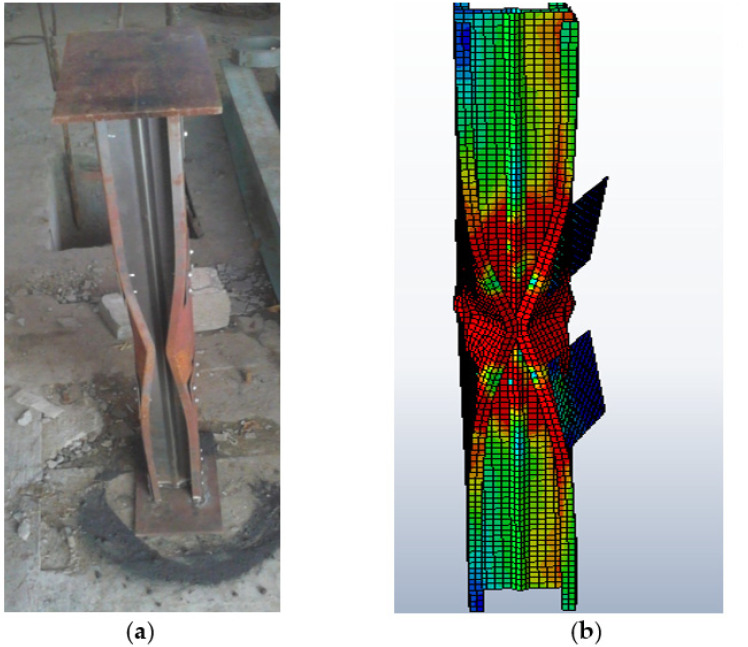
Validation of (**a**) experimental specimen tested with (**b**) the FEA model for the specimen BC-2-1.

The experimentally tested specimen was validated with FEM results, as shown in Figure 10. The test specimens were assembled in FEM, similar to the experimental setup. The screws of 8mm self-drill screws were made like the experimental models to provide the connection between the batten plate and the section. The end plate conditions are ensured to be restricted against warping due to loading. The failure load during the experiment (P_EXP_), finite element analysis (P_FEA_), and direct strength method (P_DSM_) results are tabulated in Table 4. The standard deviation and coefficient of variation of FEA to the experimental specimen are 1.072, 0.030, and 0.028. Moreover, FEA to the DSM is 0.971, 0.027, and 0.028, respectively. Therefore, from the performed FEA and experimental work, it can be inferred that using FEA, the probable buckling mode and the buckling behavior of the CFS stiffened column under experimental load subjection can be derived irrespective of slenderness ratio and geometrical specification [41].

### 3.2. Load Bearing Capacity vs. Axial Shortening Performance

The DSM approach results mentioned in Table 4 predicted the design strength under service load for the back-to-back stiffened CFS column. From the above testing, 85% of the specimen was failed by its maximum load. The column’s post-buckling behavior was enhanced by increasing axial shortening for the built-up section during the nonlinear analysis. Whatever the geometrical specification (slenderness ratio and thickens), the load increases gradually and decreases in regular intervals. The slope of the curve goes on, increasing rapidly from the initial stage.

**Figure 11:**
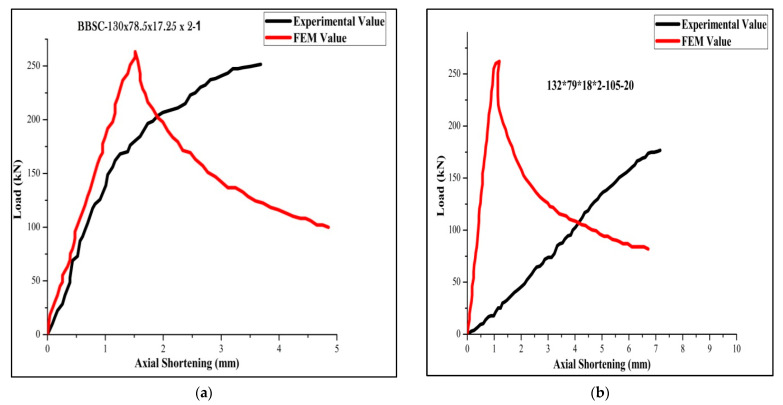
Load-versus-deflection curve for (**a**) BC 1-1 and (**b**) BC 2-2.

**Figure 12:**
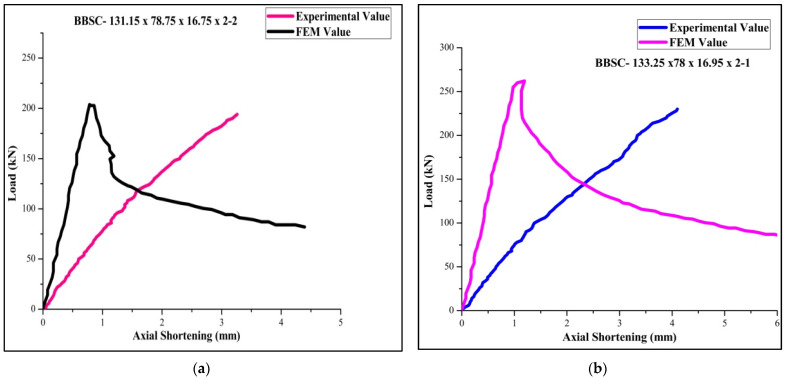
Load-versus-deflection curve for (**a**) BC 3-2 and (**b**) BC 4-1.

**Figure 13:**
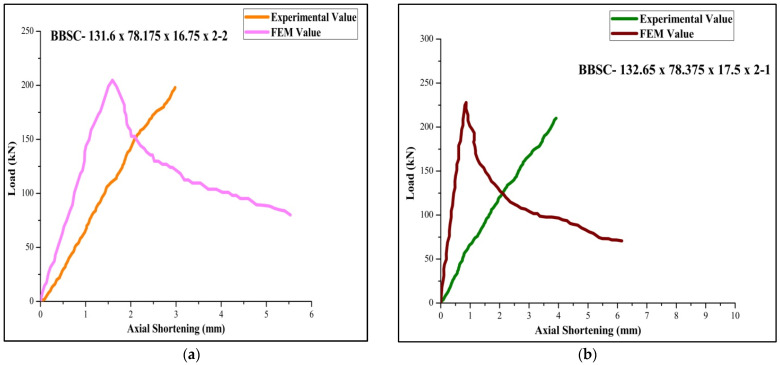
Load-versus-deflection curve for (**a**) BC 5-2 and (**b**) BC6-1.

Table 4 for the back-to-back stiffened section of 2 mm thickness for the specimen BBSC-132.5×78.75×16.75×2-1 records the maximum ultimate load of 264 kN and FEA and DSM of 273,276, respectively. The FEA results were found agreeable with the experimental results. The failure load obtained by FEA is found to be 3% more than the obtained experimental value. Moreover, obtained DSM value is found to be 2.7 % more than the predicted FEA value. Among the 36 tested specimens, 6 specimens of slenderness ratio 20 failed by local buckling and 30 specimens of slenderness ratios 30 and 40 failed by a combination of both local and distortion buckling. The load-versus-shortening curve for the boundary conditions 1, 2, 3, 4, 5, and 6 are shown in Figure 11, Figure 12 and Figure 13.

### 3.3. Comparison of DSM vs. FEM

**Figure 14:**
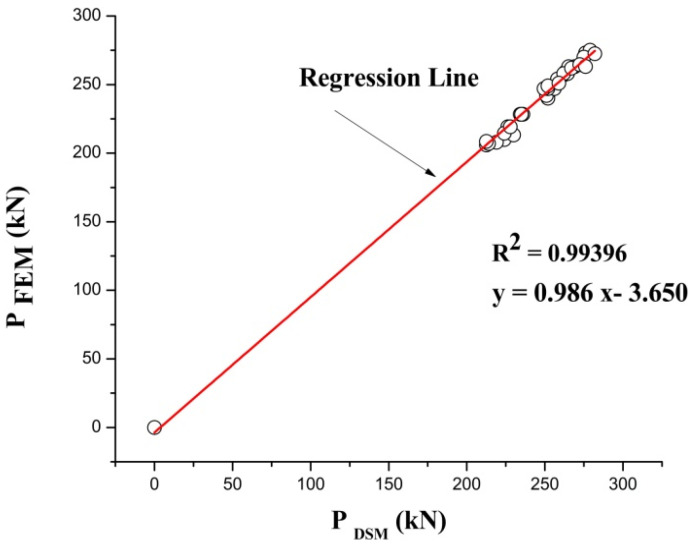
Comparison of FEM results with DSM results.

After comparing the yield load obtained from the experimentally tested model with the load availed from FEA model. The conventional DSM for predicting the failure load predicted by Muthuraman et al. [16] is adopted as a reference DSM. The failure load calculated using DSM was compared with FEM output. The comparison shows that the DSM approach was found to be a firm method for predicting the load-carrying capacity of the column. The load behavior of the DSM was found to be similar to that of the FEA predicted load. The results go on varies based on the geometrical specification. From the comparison, it is predicted that the failure load of FEM is found to be equal to 0.986 times the failure load of DSM and a difference of 3.65 (P_FEA_ = 0.986 P_DSM_ − 3.65) along with an R square value of 0.99 (1 − (Residual sum of squares/corrected Sum of squares)), as shown in Figure 14. The obtained equation can be used for all CFS stiffened built-up battened columns to predict the maximum load-carrying capacity [42,43,45].

### 3.4. Parametric Study

#### Buckling Mode

**Figure 15:**
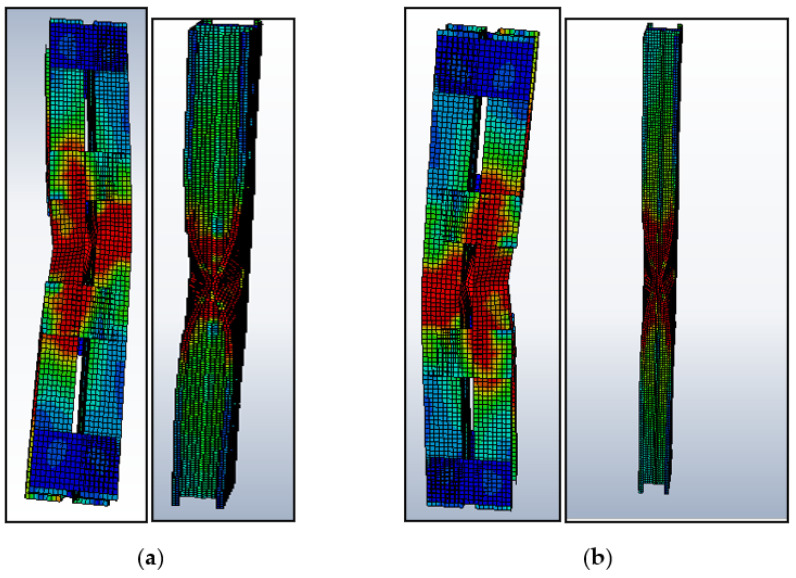
Buckling shapes obtained from FEA: (**a**) BC 1-90×60×15×2-60.27-20-4; (**b**) BC-2-90×60×15×2-120-20-4.

**Figure 16:**
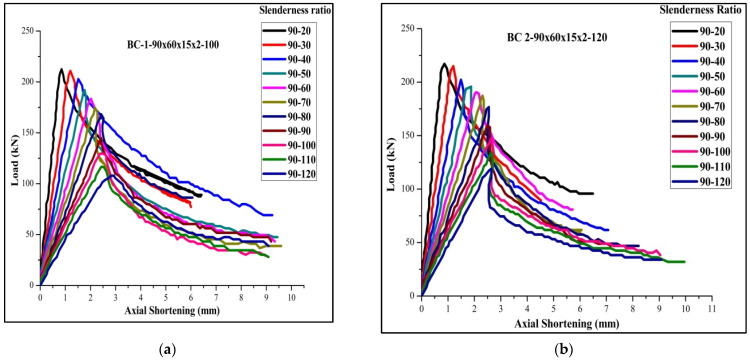
Load versus axial shortening: (**a**) boundary condition-1; (**b**) boundary condition-2.

**Figure 17:**
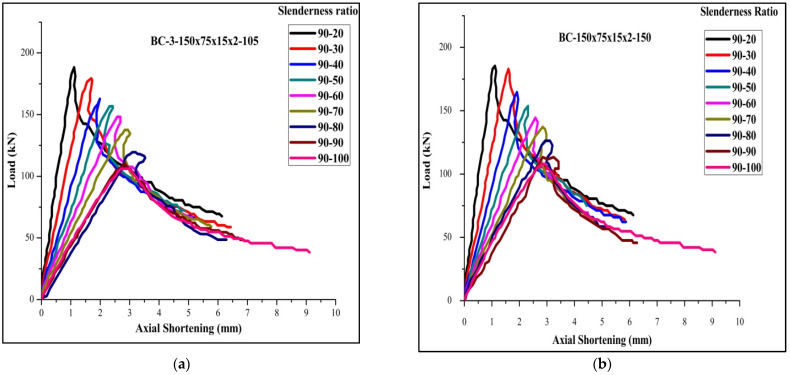
Load versus axial shortening: (**a**) boundary condition-2; (**b**) boundary condition-3.

Two different types of stiffened cross-sections were selected and varied in batten width, similar to the performed experimental work taken for parametric study. This work is detailed by studying the buckling mode of members by both analytical and theoretical (FEM and DSM) approaches. The selected section must satisfy the AISI-S100 [1] specification for the lipped stiffened section. The specification for the selected section is shown in Table 5. To find out the performance of the stiffened section, its strength obtained from analytical (P_FEM_) was compared with the load obtained theoretically (P_DSM_), and the proposed load for the experimental work can be achieved from the above-performed work in Table 4. The proposed relation shows that P_EXP_ = 0.933 times P_FEM_ is was selected as the corrected load (P_corrected_) for predicting the load using DSM. The geometrical specification of the specimen taken for the parametric study is shown in Table 5. A parametric study was carried out by varying the slenderness ratio to find out the effectiveness of the stiffener and thickness. The failure load obtained by FEA and DSM and the corrected values were compared, and the precise prediction was made from the performed work. The obtained buckling modes of the columns are shown in Figure 15. The ultimate load-carrying capacity versus axial shortening is shown in Figure 16 and Figure 17.

### 3.5. Results of Parametric Study

**Figure 18:**
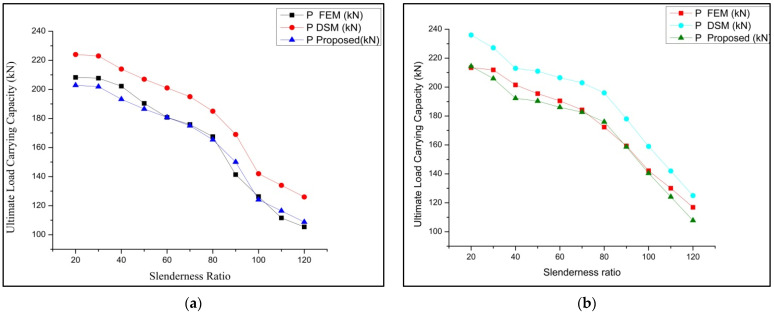
FEM versus DSM versus corrected values for (**a**) boundary condition-1 and (**b**) boundary condition-2.

**Figure 19:**
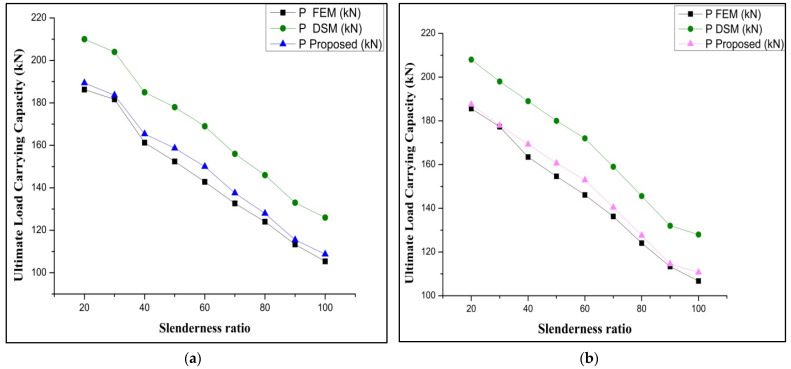
FEM versus DSM versus corrected values for (**a**) boundary condition-3 and (**b**) boundary condition-4.

**Figure 20:**
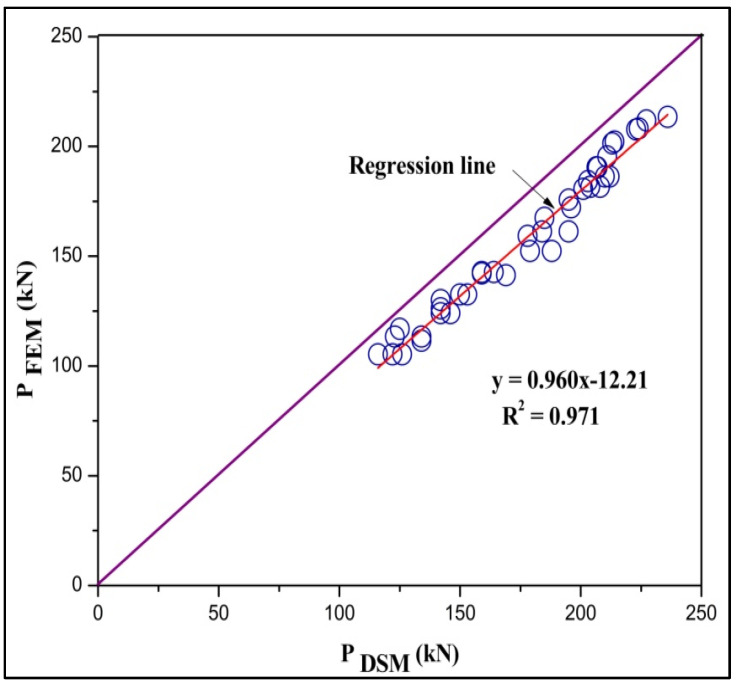
Regression analysis of P_FEA_ with P_DSM_.

**Figure 21:**
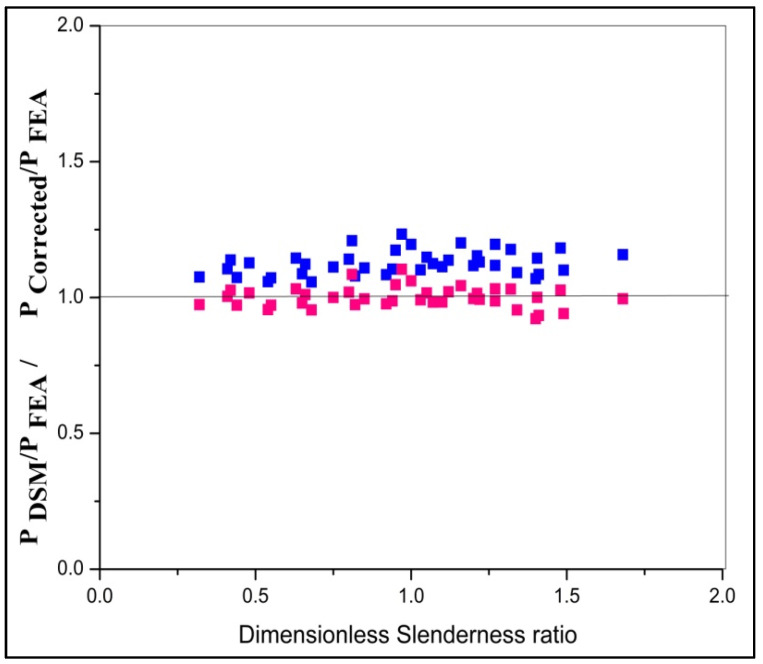
Dimensionless slenderness ratio vs. P_DSM_ and P_FEA_ values.

By FEA analysis, it can be inferred from the Table 6 that for a slenderness ratio between 20 and 60, the failure mode is the local, distortional, and flexural mode of failure (N_L_+ N_D_+ N_E_), and above 60, the failure mode is the flexural and distortional mode of failure (N_D_+ N_E_). This study enumerates the importance of failure by distortional buckling, which could influence the capacity of the chord members. From the comparison of FEA, DSM, and the corrected values, the ratio of P_DSM_ to P_FEA_ and P_Corrected values_ to P_FEA_ are 1.10 and 0.978. This method was found to reliably predict the exact failure load of DSM using FEA and obtained agreeable results, as shown in Figure 18 and Figure 19. By the regression line analysis in Figure 20, the load-carrying capacity of FEM is equal to the difference between the product of 0.96 times DSM and 12.21, and the R square value is 0.971. Therefore, this equation can be applicable for all other types of stiffened cold-formed steel built-up section columns to determine the failure load under axial loading if an analytical load is known. The load variation adapted from the following actual method P_DSM_/P_FEA_ to the corrected method P_Corrected_/P_FEA_ varies according to the slenderness ratio shown in Figure 21.

### 3.6. Discussion

From the above work, the following outcomes are discussed.

1. The experimental method shows that the load-carrying capacity is governed by residual stress, slenderness ratio, and battened width.
2. The buckling mode started with local buckling and ended up with a combination of local and distortional with respect to slenderness ratio. The buckling mode started with local buckling and ended up with the combination of local and distortional for slenderness ratio.
3. Increasing the slenderness ratio from 20 to 30 and 30 to 40, the resistance against loading decreased by the nominal verge of 10 percent for the experimental specimen. The failure of the column is predicted for a lower slenderness ratio (≤30) may be local or a combination of local and flexural buckling. For the higher-order slenderness ratio (>30), the buckling will combine local, distortional, and flexural buckling modes.
4. The experimental specimen subjected to buckling mode was matched with the FEA specimen from the validation study. Therefore, for the tedious situation, such as for the CFS back-to-back stiffened column, the observed value for a larger slenderness ratio can be predicted by finding out the product of 1.072 with P_FEM_ (analytical value).
5. From the theoretical study, it can be inferred that the DSM analysis is found to be conservative with the inclusion of service load and predicted the column’s strengthen equation, which calculates the failure load irrespective of the thickness and slenderness ratio.

## 4. Conclusions

In this study, a total of 36 specimens were examined experimentally, analytically, and theoretically to predict the load-carrying property and buckling behavior of CFS stiffened built-up columns under axial loading. From the outcomes of the validation study, a relationship was established between the FEA predicted load and DSM loads. The parametric study is carried out for the selected four different sections. The numerical investigation is performed by varying the slenderness ratio from 20 to 120 for boundary conditions 1 and 2 and 20 to 100 for boundary conditions 3 and 4. We investigated the failure load under FEA (P_FEM_), DSM (P_DSM_), and corrected values (P_corrected_) of failure load for DSM obtained using the relationship taken from the validation study. By making a complete closed section with the battened connection, investigation under axial load is considered the scope for future work.

## Figures and Tables

**Table 1:** Geometric limitation as per AISI-S100 [1] specification.

Lipped c-Section with Web Stiffener	One or Two Intermediate Stiffeners:
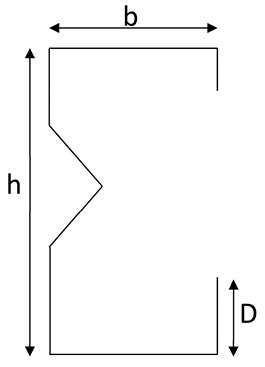	h/t < 489
	b/t < 160
	6 < D/t < 33
	1.3 < h/b < 2.7
	0.05 < D/b < 0.41
	E/*f_y_* > 340 (*f_y_* < 593 MPa or 6050 kg/cm^2^)

**Table 2:** Properties of the specimen.

No.	Specimen ID BBSC-B_W_×B_f_×D-t	Young’s Modulus E (GPa)	Tension Stress *f_y_* (MPa)	Failure Stress *f_u_* (MPa)	Elongation (%)	Loading Condition
1	BBSC-130×78.5×17.25×2	200	316	350	28	Pinned end condition
2	BBSC-132.5×78.75×16.75×2					
3	BBSC-131.15×78.75×16.75×2					
4	BBSC-131.6×78.175×16.75×2					
5	BBSC-131.6×78×16.95×2					
6	BBSC-132.65×78.375×17.5×2					

**Table 3:** Particularities of the steel specimen.

S.I.NO.	Specimen ID Boundary Condition-Specimen ID	Slenderness Ratio	Thickness t (mm)	Battened Width b (mm)	Web b_W_ (mm)	Flange b_f_ (mm)	Lip d_1_ & d_2_ (mm)	Length (mm)	Spacing between Chords S (mm)
1	BC-1-1	20	2	105	78.5	130	17.25	995	26
2	BC-2-1	30	2	105	78.5	130	17.25	1492.5	26.5
3	BC-1-2	20	2	104.5	79	130.5	17.10	995	25.2
4	BC-2-2	30	2	104.5	79	130.5	17.10	1492.5	25.7
5	BC-1-3	20	2	104.5	78.6	129.5	17.12	998	27
6	BC-2-3	30	2	104.5	78.8	129.3	17.15	1492	26.7
7	BC-3-1	20	2	149	78.75	132.5	16.75	990	27
8	BC-4-1	30	2	149	78.76	132.5	16.75	1485	26.5
9	BC-3-2	20	2	149	78.78	132.7	16.8	995	26
10	BC-4-2	30	2	149	78.32	132.6	16.65	1492	26.2
11	BC-3-3	20	2	149	77.75	132.52	16.76	1485	25
12	BC-4-3	30	2	149	78.72	132.51	16.81	1485	26.5
13	BC-5-1	30	2	105	78.75	131.15	16.75	1470	25
14	BC- 6-1	40	2	105	78.75	131.23	16.754	1960	25.6
15	BC-5-2	30	2	105	78.753	131.15	16.751	1468	24
16	BC-6-2	40	2	105	78.75	131.14	16.746	1956	23.5
17	BC-5-3	30	2	105	78.745	131.15	16.752	1469	24.2
18	BC-6-3	40	2	105	78.746	131.15	16.656	1964	24.7
19	BC-7-1	30	2	150	78.175	131.56	16.75	1472	27
20	BC-8-1	40	2	150	78.174	131.55	16.746	1964	26.25
21	BC-7-2	30	2	150	78.25	132	16.752	1462	26
22	BC-8-2	40	2	150	78.175	131.62	16.748	1949	26.5
23	BC-7-3	30	2	150	78.173	131.62	16.762	1468	25
24	BC-8-3	40	2	150	78.175	131.63	16.752	1957	25.2
25	BC-9-1	30	2	105	78	133.25	16.95	1472	27
26	BC-10-1	40	2	105	78.23	133.25	16.95	1964	26.5
27	BC-9-2	30	2	105	78.2	133.25	17	1472	26
28	BC-10-2	40	2	105	78.3	133.25	16.95	1964	26.46
29	BC-9-3	30	2	105	78.15	133.25	16.95	1472	25
30	BC-10-3	40	2	105	78	133.25	16.95	1964	27
31	BC-11-1	30	2	150	78.38	132.65	17.5	1965	29.5
32	BC-12-1	40	2	150	78.375	132.65	17.46	2620	29.62
33	BC-11-2	30	2	150	78.42	132.65	17.45	1965	28.5
34	BC-12-2	40	2	150	78.4	132.65	17.53	2620	29.25
35	BC-11-3	30	2	150	78.35	132.65	17.52	1965	31
36	BC-12-3	40	2	150	78.37	132.65	17.5	2620	32.5
30	BC-10-3	40	2	105	78	133.25	16.95	1964	27
31	BC-11-1	30	2	150	78.38	132.65	17.5	1965	29.5
32	BC-12-1	40	2	150	78.375	132.65	17.46	2620	29.62
33	BC-11-2	30	2	150	78.42	132.65	17.45	1965	28.5
34	BC-12-2	40	2	150	78.4	132.65	17.53	2620	29.25
35	BC-11-3	30	2	150	78.35	132.65	17.52	1965	31
36	BC-12-3	40	2	150	78.37	132.65	17.5	2620	32.5

**Table 4:** Comparative results of failure load in FEM (P_FEM_) with experimental (P_Exp_) and DSM (P_DSM_) results.

No.	Specimen ID Boundary Condition-Specimen ID	Slenderness Ratio	P_EXP_ (kN)	P_FEM_ (kN)	P_DSM_ (kN)	P_FEM_/P_EXP_	P_FEM_/P_DSM_
1	BC-1-1	20	250	263	269	1.052	0.978
2	BC-2-1	30	242	259	263	1.070	0.985
3	BC-1-2	20	248	261	265.3	1.052	0.984
4	BC-2-2	30	236	257.2	262.4	1.090	0.980
5	BC-1-3	20	252	263	265.2	1.044	0.992
6	BC-2-3	30	238	258	264.5	1.084	0.975
7	BC-3-1	20	264	273	276	1.034	0.989
8	BC-4-1	30	220	242	252	1.100	0.960
9	BC-3-2	20	268	275	279	1.026	0.986
10	BC-4-2	30	216	254	258	1.176	0.984
11	BC-3-3	20	258	270	275	1.047	0.982
12	BC-4-3	30	242	258	262	1.066	0.985
13	BC-5-1	30	234	244	252	1.043	0.968
14	BC-6-1	40	192	210	224	1.094	0.938
15	BC-5-2	30	230	240	252	1.043	0.952
16	BC-6-2	40	194	208	219	1.072	0.950
17	BC-5-3	30	232	242	251	1.043	0.964
18	BC-6-3	40	196	206	212.5	1.051	0.969
19	BC-7-1	30	226	247	256	1.093	0.965
20	BC-8-1	40	196	213.2	230	1.088	0.927
21	BC-7-2	30	230	247	252	1.074	0.980
22	BC-8-2	40	198	207	214	1.045	0.967
23	BC-7-3	30	228	247	249.5	1.083	0.990
24	BC-8-3	40	192	208.5	212.5	1.086	0.981
25	BC-9-1	30	230	253	257	1.100	0.984
26	BC-10-1	40	206	219.25	226.32	1.064	0.969
27	BC-9-2	30	226	249	252	1.102	0.988
28	BC-10-2	40	206	214.5	224	1.041	0.958
29	BC-9-3	30	236	251	259	1.064	0.969
30	BC-10-3	40	206	219.25	227.89	1.064	0.962
31	BC-11-1	30	250	272.5	282	1.090	0.966
32	BC-12-1	40	210	228.33	236	1.087	0.968
33	BC-11-2	30	242	264.5	272.4	1.093	0.971
34	BC-12-2	40	210	228.33	234.23	1.087	0.975
35	BC-11-3	30	246	263.2	276	1.070	0.954
36	BC-12-3	40	210	228.33	235	1.087	0.972
		Mean				1.072	0.971
		Standard Deviation				0.030	0.027
		Coefficient of Variation				0.028	0.025

**Table 5:** Properties of the chosen section for parametric study.

No.	Specimen ID BC 1-B_W_×B_f_× D-t-b_w_	Batten Width (mm)	Thickness (mm)	Spacing between Chords (mm)	Ultimate Strength f_u_ (N/mm^2^) and Poisson’s Ratio	Slenderness Ratio	Young’s Modulus (N/mm^2^)
1	BC 1-90×60×15-2-60.27	60.27	2	25	305 and 0.3	20-120	2 × 10^5^
2	BC 2-90×60×15-2-120	120	2	25		20-120	
3	BC 3-150×75×15-2-150	150	2	73		20-100	
4	BC 4-150×75×15-2-105	105	2	73		20-100	

**Table 6:** Results of parametric study and comparison of FEM failure load versus DSM and corrected values.

No.	Specimen ID	Slenderness Ratio	P_FEM_ (KN)	P_DSM_ (kN)	P_Corrected_ (kN)	P_Corrected_ /P_FEM_	P_DSM_ /P_FEM_	Failure Mode
1	BC 1-90×60×15×2-60.27-20-4	20	208.250	224.000	202.830	0.974	1.076	N_L_ + N_D_ + N_E_
2	BC 1-90×60×15×2-60.27-30-5	30	207.719	223.000	201.870	0.972	1.074	N_L_ + N_D_ + N_E_
3	BC 1-90×60×15×2-60.27-40-5	40	202.221	214.000	193.230	0.956	1.058	N_L_ + N_D_ + N_E_
4	BC 1-90×60×15×2-60.27-50-5	50	190.381	207.000	186.510	0.980	1.087	N_L_ + N_D_ + N_E_
5	BC 1-90×60×15×2-60.27-60-6	60	180.738	201.000	180.750	1.000	1.112	N_L_ + N_D_ + N_E_
6	BC 1-90×60×15×2-60.27-70-6	70	175.848	195.000	174.990	0.995	1.109	N_D_ + N_E_
7	BC 1-90×60×15×2-60.27-80-7	80	167.495	185.000	165.390	0.987	1.105	N_D_ + N_E_
8	BC 1-90×60×15×2-60.27-90-8	90	141.358	169.000	150.030	1.061	1.196	N_D_ + N_E_
9	BC 1-90×60×15×2-60.27-100-9	100	126.266	142.000	124.110	0.983	1.125	N_D_ + N_E_
10	BC 1-90×60×15×2-60.27-110-10	110	111.572	134.000	116.430	1.044	1.201	N_D_ + N_E_
11	BC 1-90×60×15×2-60.27-120-10	120	105.377	126.000	108.750	1.032	1.196	N_D_ + N_E_
12	BC 2-90×60×15×2-120-20-4	20	213.432	236.000	214.350	1.004	1.106	N_L_ + N_D_ + N_E_
13	BC 2-90×60×15×2-120-30-5	30	211.901	227.200	205.902	0.972	1.072	N_L_ + N_D_ + N_E_
14	BC 2-90×60×15×2-120-40-5	40	201.476	213.000	192.270	0.954	1.057	N_L_ + N_D_ + N_E_
15	BC 2-90×60×15×2-120-50-5	50	195.485	211.000	190.350	0.974	1.079	N_L_ + N_D_ + N_E_
16	BC 2-90×60×15×2-120-60-6	60	190.488	206.500	186.030	0.977	1.084	N_L_ + N_D_ + N_E_
17	BC 2-90×60×15×2-120-70-6	70	184.242	203.000	182.670	0.991	1.102	N_D_ + N_E_
18	BC 2-90×60×15×2-120-80-7	80	172.343	196.000	175.950	1.021	1.137	N_D_ + N_E_
19	BC 2-90×60×15×2-120-90-8	90	159.321	178.000	158.670	0.996	1.117	N_D_ + N_E_
20	BC 2-90×60×15×2-120-100-9	100	142.187	159.000	140.430	0.988	1.118	N_D_ + N_E_
21	BC 2-90×60×15×2-120-110-10	110	130.060	142.000	124.110	0.954	1.092	N_D_ + N_E_
22	BC 2-90×60×15×2-120-120-10	120	116.872	125.000	107.790	0.922	1.070	N_D_ + N_E_
23	BC 3-150×75×15-2-20-150-5	20	186.273	210.000	189.390	1.017	1.127	N_L_ + N_D_ + N_E_
24	BC 3-150×75×15-2-20-150-5	30	181.683	204.000	183.630	1.011	1.123	N_L_ + N_D_ + N_E_
25	BC 3-150×75×15-2-20-150-5	40	161.259	185.000	165.390	1.026	1.147	N_L_ + N_D_ + N_E_
26	BC 3-150×75×15-2-20-150-6	50	152.433	178.000	158.670	1.041	1.168	N_L_ + N_D_ + N_E_
27	BC 3-150×75×15-2-20-150-7	60	142.812	169.000	150.030	1.051	1.183	N_L_ + N_D_ + N_E_
28	BC 3-150×75×15-2-20-150-8	70	132.662	156.000	137.550	1.037	1.176	N_D_ + N_E_
29	BC 3-150×75×15-2-20-150-10	80	124.053	146.000	127.950	1.031	1.177	N_D_ + N_E_
30	BC 3-150×75×15-2-20-150-11	90	113.355	133.000	115.470	1.019	1.173	N_D_ + N_E_
31	BC 3-150×75×15-2-20-150-12	100	105.377	126.000	108.750	1.032	1.196	N_D_ + N_E_
32	BC 4-150×75×15-2-20-105-5	20	185.596	208.000	187.470	1.010	1.121	N_L_ + N_D_ + N_E_
33	BC 4-150×75×15-2-20-105-5	30	177.289	198.000	177.870	1.003	1.117	N_L_ + N_D_ + N_E_
34	BC 4-150×75×15-2-20-105-5	40	163.444	189.000	169.230	1.035	1.156	N_L_ + N_D_ + N_E_
35	BC 4-150×75×15-2-20-105-6	50	154.630	180.000	160.590	1.039	1.164	N_L_ + N_D_ + N_E_
36	BC 4-150×75×15-2-20-105-7	60	146.100	172.000	152.910	1.047	1.177	N_L_ + N_D_ + N_E_
37	BC 4-150×75×15-2-20-105-8	70	136.274	159.000	140.430	1.030	1.167	N_D_ + N_E_
38	BC 4-150×75×15-2-20-105-10	80	124.053	145.600	127.566	1.028	1.174	N_D_ + N_E_
39	BC 4-150×75×15-2-20-105-11	90	113.355	132.000	114.510	1.010	1.164	N_D_ + N_E_
40	BC 4-150×75×15-2-20-105-12	100	106.743	128.000	110.670	1.037	1.199	N_D_ + N_E_
Mean						0.978	1.101	
Standard Deviation						0.027	0.031	
Coefficient of Variation						0.028	0.028	

Annotations: N_L_—Local Buckling; N_D_—Distortion Buckling; N_E_—Flexural Buckling.

## Data Availability

Data sharing not applicable.

## References

[1] (2007). North American Specification for the Design of Cold-Formed Steel Structural Members Specifications.

[2] Salem A., Aghoury M., Dib F.H., Hanna M. (2004). Ultimate capacity of I-slender section columns. J. Constr. Steel Res..

[3] Kalochairetis K., Gantes C. (2011). Numerical and analytical investigation of collapse loads of laced built-up columns. Comput. Struct..

[4] Kandasamy R., Thenmozhi R., Jayagopal L. (2015). Flexural -Torsional Buckling Tests of Cold-Formed Lipped Channel Beams Under Restrained Boundary Conditions. KSCE J. Civ. Eng..

[5] Rondal J., Niazi M. (1990). Stability of built-up beams and columns with thin-walled members. J. Constr. Steel Res..

[6] Aslani F., Goel S.C. (1991). An Analytical Criterion for Buckling Strength of Built-up Compression Members. Eng. J..

[7] Roy K., Ting T., Lau H.H., Lim J. (2018). Nonlinear behavior of axially loaded back-to-back built-up cold-formed steel un-lipped channel sections. Steel Compos. Struct..

[8] Roy K., Ting T., Lau H.H., Lim J. (2018). Experimental investigation into the behaviour of back-to-back gapped built-up cold-formed steel channel sections under compression. J. Constr. Steel Res..

[9] Roy K., Ting T., Lau H.H., Lim J. (2018). Effect of thickness on the behaviour of axially loaded back-to-back cold-formed steel built-up channel sections -Experimental and numerical investigation. Structures..

[10] (2005). Australian/New Zealand Standard—Cold Formed Steel Structures.

[11] Sani M.S.H.M., Muftah F., Tan C.S. (2020). Experimental Analysis of Cold-Formed Steel C-Sections with the Notch Subjected to Axial Compression. KSCE J. Civ. Eng..

[12] (2006). Eurocode 3- Design of Steel Structures—Part 1-3: General Rules—Supplementary Rules for Cold-Formed Members and Sheeting vol. Part 1-3. https://standards.iteh.ai/catalog/standards/cen/2f1f2ecc-0d2a-481d-a432-9d833ea1861f/en-1993-1-3-2006.

[13] Anbarasu M., Kumar S.B., Sukumar S. (2013). Study on the effect of ties in the intermediate length Cold Formed Steel (CFS) columns. Struct. Eng. Mech..

[14] el Aghoury M.A., Salem A.H., Hanna M.T., Amoush E.A. (2013). Ultimate capacity of battened columns composed of four equal slender angles. Thin-Walled Struct..

[15] el Aghoury M.A., Salem A.H., Hanna M.T., Amoush E.A. (2010). Experimental investigation for the behaviour of battened beam-columns composed of four equal slender angles. Thin-Walled Struct..

[16] Georgieva I., Schueremans L., Pyl L. (2012). Composed columns from cold-formed steel Z-profiles: Experiments and code-based predictions of the overall compression capacity. Eng. Struct..

[17] Georgieva I., Schueremans L., Pyl L., Vandewalle L. (2012). Experimental investigation of built-up double-Z members in bending and compression. Thin-Walled Struct..

[18] Anbarasu M., Kanagarasu K., Sukumar S. (2015). Investigation on the behaviour and strength of cold-formed steel web stiffened built-up battened columns. Mater. Struct..

[19] Muthuraman M., Anuradha R., Awoyera P.O., Gobinath R. (2020). Numerical simulation and specification provisions for buckling characteristics of a built-up steel column section subjected to axial loading. Eng. Struct..

[20] Ting C.H.T., Lau H.H. (2011). Compression Test on Cold-Formed Steel Built-Up Back-to-Back Channels Stub Columns. Adv. Mater. Res..

[21] Schafer B.W., Peköz T. (1998). Computational modeling of cold-formed steel: Characterizing geometric imperfections and residual stresses. J. Constr. Steel Res..

[22] Kankanamge N.D., Mahendran M. (2012). Behaviour and design of cold-formed steel beams subject to lateral–torsional buckling. Thin-Walled Struct..

[23] Ye J., Hajirasouliha I., Becque J., Eslami A. (2016). Optimum design of cold-formed steel beams using Particle Swarm Optimisation method. J. Constr. Steel Res..

[24] Dinis P.B., Camotim D. (2015). Cold-formed steel columns undergoing local–distortional coupling: Behaviour and direct strength prediction against interactive failure. Comput. Struct..

[25] Ye J., Hajirasouliha I., Becque J. (2018). Experimental investigation of local-flexural interactive buckling of cold-formed steel channel columns. Thin-Walled Struct..

[26] Aswathy K.C.K., Kumar M.v.A. (2020). Unstiffened Elements as Limiting Case of Distortional Buckling of Partially Stiffened Elements. J. Struct. Eng..

[27] Kumar M.v.A., Kalyanaraman V. (2018). Interaction of Local, Distortional, and Global Buckling in CFS Lipped Channel Compression Members. J. Struct. Eng..

[28] Dar M.A., Sahoo D.R., Jain A.K. (2020). Numerical Study on the Structural Integrity of Built-up Cold-Formed Steel Battened Columns.

[29] Kherbouche S., Megnounif A. (2019). Numerical study and design of thin walled cold formed steel built-up open and closed section columns. Eng. Struct..

[30] Dar M.A., Sahoo D.R., Jain A.K. (2020). Influence of chord compactness and slenderness on axial compression behavior of built-up battened CFS columns. J. Build. Eng..

[31] Vijayanand S., Anbarasu M. (2017). Effect of Spacers on Ultimate Strength and Behavior of Cold-Formed Steel Built-up Columns. Procedia Eng..

[32] Anbarasu M. (2020). Behaviour of cold-formed steel built-up battened columns composed of four lipped angles: Tests and numerical validation. Adv. Struct. Eng..

[33] Zhang J.-H., Young B. (2018). Finite element analysis and design of cold-formed steel built-up closed section columns with web stiffeners. Thin-Walled Struct..

[34] Gunalan S., Mahendran M. (2013). Improved design rules for fixed ended cold-formed steel columns subject to flexural–torsional buckling. Thin-Walled Struct..

[35] Martins A.D., Dinis P.B., Camotim D., Providência P. (2015). On the relevance of local–distortional interaction effects in the behaviour and design of cold-formed steel columns. Comput. Struct..

[36] Cava D., Camotim D., Dinis P.B., Madeo A. (2016). Numerical investigation and direct strength design of cold-formed steel lipped channel columns experiencing local–distortional–global interaction. Thin-Walled Struct..

[37] Manikandan P., Arun N. (2016). Behaviour of Partially Closed Stiffened Cold-Formed Steel Compression Member. Arab. J. Sci. Eng..

[38] Fang Z., Roy K., Mares J., Sham C.-W., Chen B., Lim J.B.P. (2021). Deep learning-based axial capacity prediction for cold-formed steel channel sections using Deep Belief Network. Structures.

[39] Taheri E., Fard S.E., Zandi Y., Samali B. (2021). Experimental and Numerical Investigation of an Innovative Method for Strengthening Cold-Formed Steel Profiles in Bending throughout Finite Element Modeling and Application of Neural Network Based on Feature Selection Method. Appl. Sci..

[40] Kanthasamy E., Alsanat H., Poologanathan K., Gatheeshgar P., Corradi M., Thirunavukkarasu K., Dissanayake M. (2022). Web Crippling Behaviour of Cold-Formed High Strength Steel Unlipped Channel Beams. Buildings.

[41] Roy K., Lau H.H., Fang Z., Masood R., Ting T.C., Lim J.B., Lee V.C. (2022). Effects of corrosion on the strength of self-drilling screw connections in cold-formed steel structures-experiments and finite element modeling. Structures.

[42] Liang H., Roy K., Fang Z., Lim J.B.P. (2022). A Critical Review on Optimization of Cold-Formed Steel Members for Better Structural and Thermal Performances. Buildings.

[43] Anbarasu M., Murugapandian G. (2016). Experimental study on cold-formed steel web stiffened lipped channel columns undergoing distortional–global interaction. Mater. Struct..

[44] (2010). ABAQUS (2010), Version 6.10.

[45] (1996). Standard Test Methods and Definitions for Mechanical Testing of Steel Products.

